# Agreement between primary care and hospital diagnosis of schizophrenia and bipolar disorder: A cross-sectional, observational study using record linkage

**DOI:** 10.1371/journal.pone.0210214

**Published:** 2019-01-07

**Authors:** Braden O’Neill, Sumeet Kalia, Babak Aliarzadeh, Rahim Moineddin, Wai Lun Alan Fung, Frank Sullivan, Asmaa Maloul, Steven Bernard, Michelle Greiver

**Affiliations:** 1 Department of Family and Community Medicine, North York General Hospital, Toronto, Canada; 2 Department of Family and Community Medicine, Faculty of Medicine, University of Toronto, Toronto, Canada; 3 University of Toronto Practice-Based Research Network, University of Toronto, Toronto, Canada; 4 Research and Innovation, North York General Hospital, Toronto, Canada; 5 Department of Psychiatry, North York General Hospital, Toronto, Canada; 6 Department of Psychiatry, Faculty of Medicine, University of Toronto, Toronto, Canada; 7 School of Medicine, University of St. Andrews, St. Andrews, Scotland; 8 North York General Hospital, Toronto, Canada; Department of Psychiatry and Neuropsychology, Maastricht University Medical Center, NETHERLANDS

## Abstract

People with serious mental illness die 10–25 years sooner than people without these conditions. Multiple challenges to accessing and benefitting from healthcare have been identified amongst this population, including a lack of coordination between mental health services and general health services. It has been identified in other conditions such as diabetes that accurate documentation of diagnosis in the primary care chart is associated with better quality of care. It is suspected that if a patient admitted to the hospital with serious mental illness is then discharged without adequate identification of their diagnosis in the primary care setting, follow up (such as medication management and care coordination) may be more difficult. We identified cohorts of patients with schizophrenia and bipolar disorder who accessed care through the North York Family Health Team (a group of 77 family physicians in Toronto, Canada) and North York General Hospital (a large community hospital) between January 1, 2012 and December 31, 2014. We identified whether labeling for these conditions was concordant between the two settings and explored predictors of concordant labeling. This was a retrospective cross-sectional study using de-identified data from the Health Databank Collaborative, a linked primary care-hospital database. We identified 168 patients with schizophrenia and 370 patients with bipolar disorder. Overall diagnostic concordance between primary care and hospital records was 23.2% for schizophrenia and 15.7% for bipolar disorder. Concordance was higher for those with multiple (2+) inpatient visits (for schizophrenia: OR 2.42; 95% CI 0.64–9.20 and for bipolar disorder: OR 8.38; 95% CI 3.16–22.22). Capture-recapture modeling estimated that 37.4% of patients with schizophrenia (95% CI 20.7–54.1) and 39.6% with bipolar disorder (95% CI 25.7–53.6) had missing labels in both settings when adjusting for patients’ age, sex, income quintiles and co-morbidities. In this sample of patients accessing care at a large family health team and community hospital, concordance of diagnostic information about serious mental illness was low. Interventions should be developed to improve diagnosis and continuity of care across multiple settings.

## Introduction

Schizophrenia and bipolar disorder are serious mental illnesses (SMIs) affecting approximately 1% of the population [1–2]. These conditions are associated with a substantially increased risk of early death, primarily from cardiovascular disease and neoplasms [3]. People with these conditions die 10–25 years sooner than those without with most of the early mortality related to cardiovascular morbidity rather than mental health issues [3–4]. They frequently have high degrees of comorbidities and are cared for by different health care providers including family physicians and mental health professionals [5]. Those with SMI have more frequent hospital admissions, both for mental health and physical health related reasons [6].

Accurate and complete documentation of conditions is essential to allow health care providers to recognize whether any patient lives with a condition. Accurate documentation also allows generation of practice cohorts; this can lead to identification of risks relevant to all patients with a condition as well as planning interventions to address these [7]. In primary care, the ‘cumulative patient profile’ constitutes the definitive source of information regarding health conditions for patients. It is considered the “master list” for health information for patients by regulatory authorities [8]. If a condition is not present in this list, specific risks associated with that condition may not be identified or acted upon. Patients with SMIs experience high mortality and substantial morbidity from conditions that are amenable to primary prevention in primary care, such as cardiovascular disease and cancer. It is critical that SMIs be documented appropriately in the primary care record: this will allow identification of practice populations at elevated risk, planning of strategies to address risk and measurement and improvement of quality of care [9].

Patients with SMIs frequently access care in both hospitals and primary care. They are more likely to be hospitalized than those without SMIs [10]. Upon discharge from hospital, these patients may access follow up care with psychiatrists, other mental health professionals and family physicians [11]. Family physicians are most commonly tasked with helping patients with SMI manage their physical health; factors associated with cardiovascular health are the most substantial contributors to early mortality [12].

In Ontario, Canada’s largest province, integrated care systems do not exist between primary care and hospital services. However, there are high degrees of loyalty in referral networks from local primary care providers to hospital services, as well as in patients who access care from local primary care providers attending the local hospital emergency department preferentially when necessary. This geographic loyalty is estimated at 70%, which means that most of the clinical data for patients are held between their primary care provider and their local hospital [13].

In order to understand primary and secondary care use, diagnostic discrepancies, and other important issues in the community that they jointly serve, the North York General Hospital (NYGH), a large community academic hospital in Toronto, Ontario, Canada and the North York Family Health Team (NYFHT), a primary care organization including family physicians and allied health professionals serving > 100 000 people, developed a linked primary-secondary care database called the Health Databank Collaborative (HDC) [14]. This database includes almost all data from emergency department and hospital records (other than data from inpatient mental health admissions) and participating primary care physicians’ data from the University of Toronto Practice-Based Research Network (UTOPIAN) database [5]. By linking these two comprehensive data sources and creating a combined database, it became feasible to explore patterns of care for patients seen in both settings, such as those with SMIs.

Our aims were to identify a cohort of patients labeled as having schizophrenia and bipolar disorder, who had been seen in both settings (primary care and hospital), to estimate labeling agreement, and to determine patient factors associated with labeling agreement. We also aimed to estimate the size of the populations of interest through capture-recapture modeling, a statistical approach that facilitates estimation of population size from multiple samples taken from the same population.

## Methods

### Data sources

The study was reviewed and approved by the North York General Hospital (NYGH) Research Ethics Board (REB). Data collection through the University of Toronto Practice-Based Research Network (UTOPIAN) has been approved by the REB at the University of Toronto, as well as NYGH. All data were fully anonymized prior to analysis. We conducted a retrospective, cross-sectional study using the HDC database, which contains data from two sources: NYFHT physicians’ electronic medical records (primary care) and from NYGH (hospital records). Primary care data obtained from the NYFHT are extracted, standardized, merged and stored as part of the routine activities of the University of Toronto Practice-Based Research Network (UTOPIAN), one of 11 practice-based research networks (PBRNs) that comprise the Canadian Primary Care Sentinel Surveillance Network (CPCSSN) [15]. All of these PBRNs use similar data extraction and management processes [16]. Participating family physicians and other primary care providers contribute de-identified data from their patients to the UTOPIAN data safe haven. Patients are informed about this through posters in clinic waiting rooms and are able to opt-out. In the HDC, primary care data is linked to secondary care data by NYGH analyst using a one-way encrypted unique identifier, derived from provincial health card number for each patient [14]. We conducted our analyses on datasets generated from the combined database.

For hospital data, we used the Canadian Institute for Health Information National Ambulatory Care Reporting System (CIHI NACRS; for emergency department visits) [17] and Canadian Institute for Health Information Discharge Abstract Database (CIHI DAD; for medical inpatient visits) [18] to determine whether a SMI diagnosis was present as part of a hospital encounter. CIHI is an independent, not-for-profit organization that provides essential information on Canada’s health systems and the health of Canadians, for multiple purposes including health systems planning and research. Trained CIHI abstractors review and enter standardized visit information for NACRS and DAD. Mental health inpatient hospital visits in Ontario are also recorded in the Ontario Mental Health Reporting System (OMHRS) [19]. CIHI DAD includes information from all hospitalizations excluding inpatient mental health visits; specific information about these visits is captured in OMHRS. These OMHRS data incur restricted access and were not included in the HDC; however since almost all inpatient admissions to mental health at NYGH come through the emergency department (from which data for admission diagnoses and co-morbidities is available through NACRS coding) we were able to capture relevant interactions with the hospital services.

### Cohort generation

**Fig 1:**
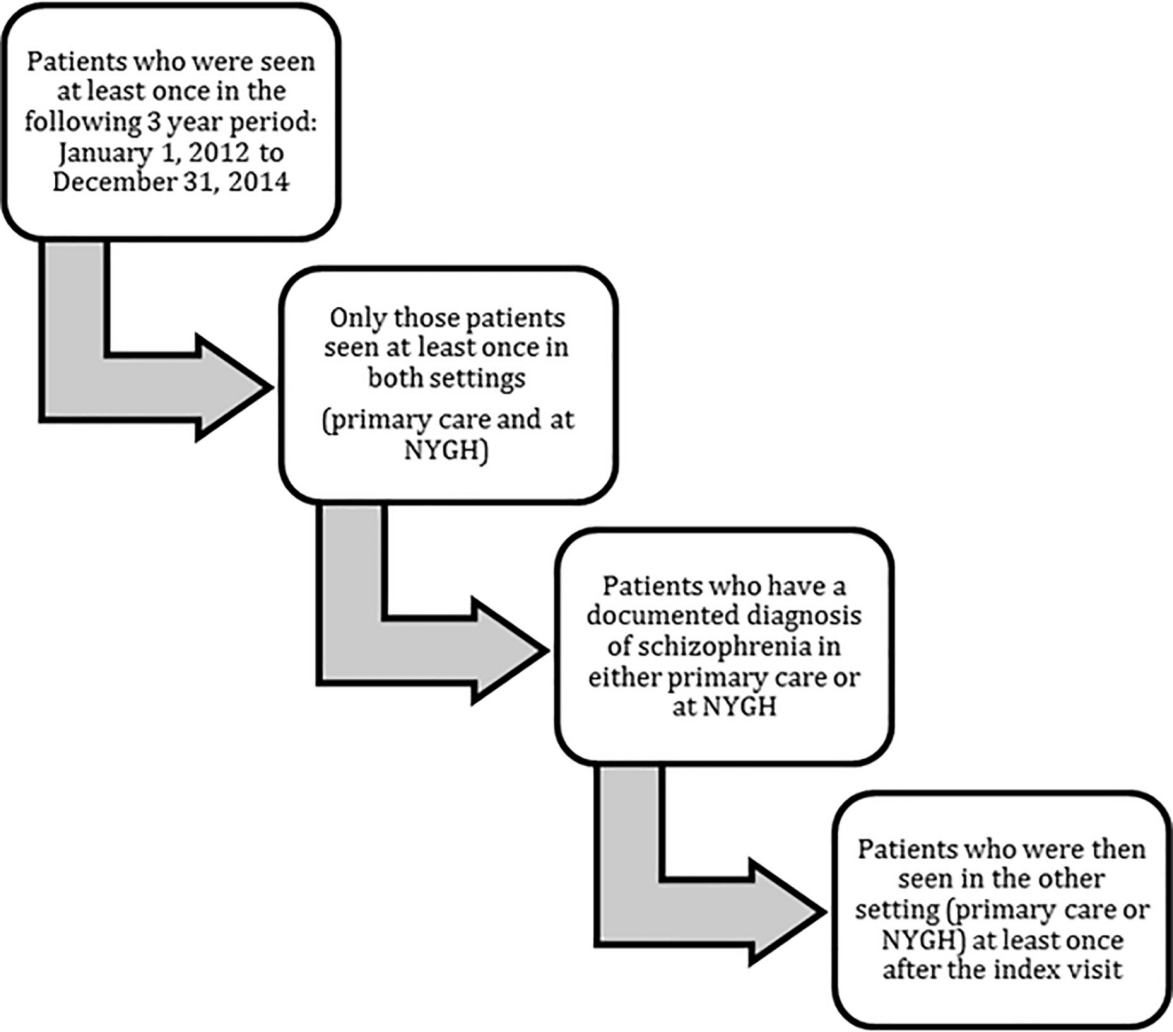
Generation of schizophrenia cohort.

All patients who had accessed care at least once in both settings (hospital and primary care) during a 3 year period from 1 January 2012–31 December 2014 were included in the analysis. The specification of this study period allowed us to compare recent results with an earlier study where we evaluated the diagnostic labels for COPD (chronic obstructive pulmonary disease) and HF (heart failure) conditions using the same data source and a similar methodology [20]. A diagnostic label of schizophrenia or bipolar disorder (or related terms, see below) must have been present in at least one setting (primary care or hospital records) at any time prior to 31 December 2014. There must have been one or more visits to the primary care provider following the hospital visit where schizophrenia or bipolar disorder was initially recorded, or one hospital visit after schizophrenia or bipolar disorder was recorded in the primary care chart. The generation of the cohort for schizophrenia is described in Fig 1. A similar process was used for bipolar disorder.

For hospital data, we included all patients whose visits included ICD-10 codes were consistent with the conditions of interest: for 'schizophrenia spectrum disorders' schizophrenia, ICD-10 F20 (schizophrenia), F25 (schizoaffective disorder), and F29 (psychosis not otherwise specified). This definition was chosen because it has been established as a sensitive definition in Ontario administrative data [21]. We refer to these conditions collectively as “schizophrenia” in the rest of the paper. For bipolar disorder, we included ICD-10 F31 (bipolar disorder).

In primary care data, we included ICD-9 295 (schizophrenia) and 298 (psychosis not otherwise specified), as well as free text search for “%PSYCHOS%” or “%SCHIZOPHREN%” key words in the cumulative patient profile, billing data, and encounter diagnoses. We excluded any free text diagnosis recorded as family history or associated with negating words or any words that makes the diagnosis uncertain (i.e. “evaluate for schizophrenia”, “father had psychosis”). This query was extensively tested and generated samples of outputs were manually reviewed to ascertain its accuracy. We chose this definition to maximize sensitivity, and because it is again consistent with that identified for Ontario administrative data [21]. In order to increase specificity, we developed a series of exclusions, which we ran as part of the search (such as to exclude “unspecified psychological condition” from the search using the free text term “%psycho%”). For bipolar disorder, we included ICD-9 296 or presence of the free text term “bipolar” in the cumulative patient profile.

### Data extraction

We extracted the following data elements from the HDC database: patient age as of 31 December 2014, patient sex, socioeconomic quintiles (determined by geographically derived information based on Statistics Canada’s Postal Code Conversion File) [22–23]. We identified presence of co-morbidities, based on 8 previously validated definitions for chronic conditions in CPCSSN [24]: diabetes, hypertension, osteoarthritis, depression, COPD, dementia, epilepsy, and Parkinson’s disease. We also measured health care utilization with the following metrics: primary care visits, hospital utilization. Hospital utilization was determined by the number of emergency department visits, and the number of inpatient admissions. If there was an emergency department visit followed by an inpatient admission, this was counted as one inpatient admission.

### Statistical analyses

All analyses were conducted using SAS version 9.4. We recorded the extent to which labeling for schizophrenia and bipolar disorder was present in primary care records, and in hospital records. We determined the proportion of patients with concordant labeling in both settings, versus those with labels in only one setting. Associations between concordant labeling and clinical factors were explored using logistic regression. These associations were reported with adjusted odds ratios and 95% confidence intervals. We used capture-recapture modelling to estimate the total size of the population of people accessing care between NYGH and NYFHT who have the conditions of interest. This involves estimation of the probability that patients have the condition of interest, were seen in both settings, and had their diagnostic label missed by both. This approach was originally developed in biological studies to estimate the population size of a mobile population; it can be applied in epidemiology where there are multiple lists of patients with some overlap between them [25]. Further details are available in S1 Supporting Information.

The study was reviewed and approved by the NYGH Research Ethics Board (REB). Data collection through UTOPIAN has been approved by the REB at the University of Toronto, as well as NYGH. All primary care providers whose data are included in this study have provided written informed consent for its collection and analysis. We followed the STROBE guidelines for reporting observational studies [26].

## Results

**Fig 2:**
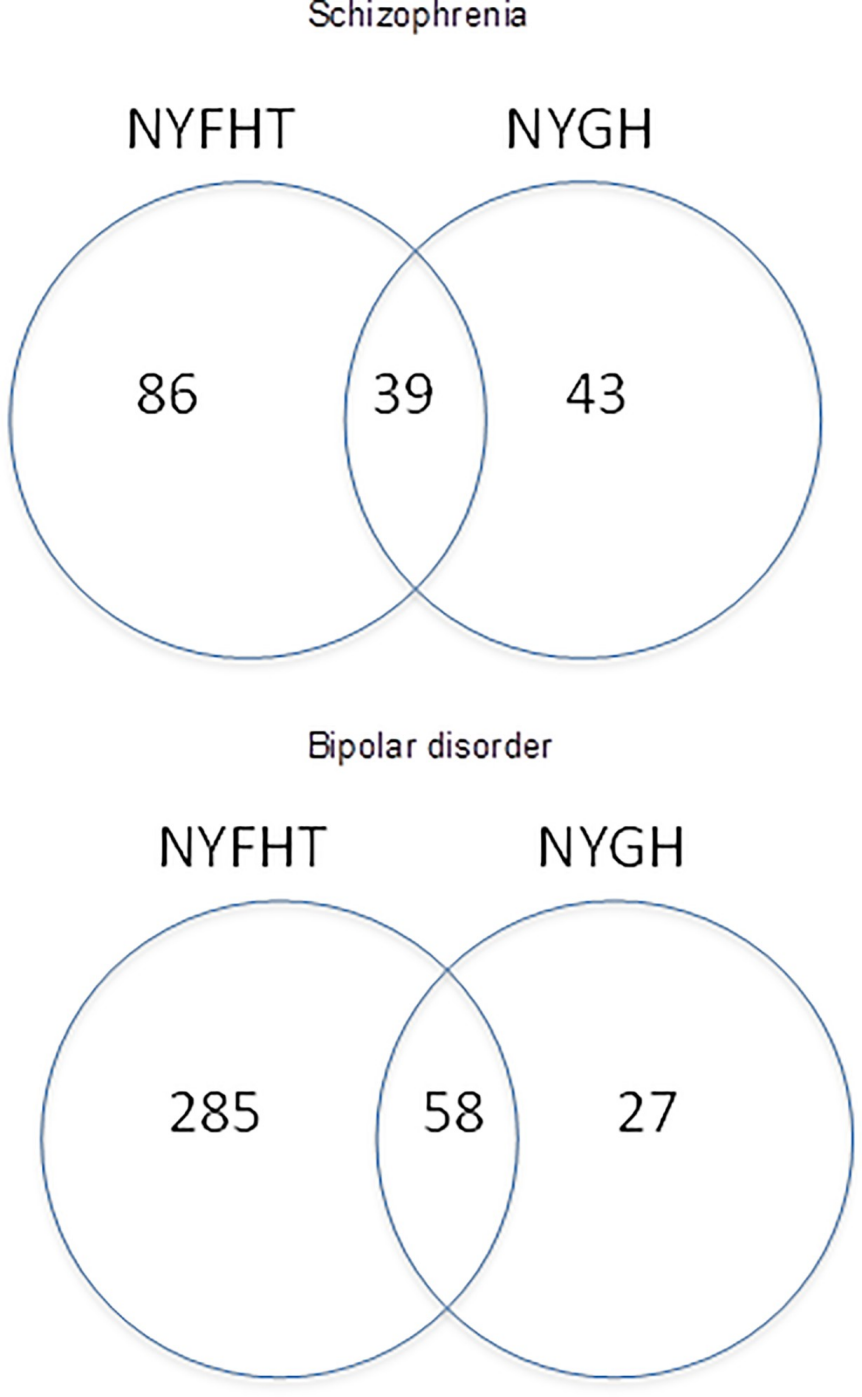
Location of diagnostic information.

77 family physicians provided data to the HDC, with 103,577 patients age 16 or more enrolled to these physicians on 31 December 2014. Tables 1 and 2 show patient characteristics for schizophrenia and bipolar disorder, respectively. Fig 2 shows the location of diagnostic information for both conditions. The results of regression modeling for concordant labeling in the two settings are shown in Table 3 for both conditions.

**Table 1:** Characteristics of patients with schizophrenia identified in primary care (NYFHT) and hospital (NYGH) data, and those identified in both settings.

Patient characteristics	NYFHT		NYGH		NYGH and NYFHT		Total
	N	Percentage (%)	N	Percentage (%)	N	Percentage (%)	N
**Age range (years)**	26	41.9%	19	30.6%	17	27.4%	62
16–40							
41–60	25	48.1%	12	23.1%	15	28.8%	52
61+	35	64.8%	12	22.2%	7	13.0%	54
**Deceased**	86	51.8%	41	24.7%	39	23.5%	166
No							
Yes	.	.	2	100.0%	.	.	2
**Gender**	49	50.5%	27	27.8%	21	21.6%	97
F							
M	37	52.1%	16	22.5%	18	25.4%	71
**Number of co-morbidities**	51	46.4%	31	28.2%	28	25.5%	110
0–1							
2+	35	60.3%	12	20.7%	11	19.0%	58
**Income quintiles**	7	29.2%	11	45.8%	6	25.0%	24
Missing							
1 (= lowest)	26	60.5%	5	11.6%	12	27.9%	43
2	9	56.3%	3	18.8%	4	25.0%	16
3	16	69.6%	2	8.7%	5	21.7%	23
4	11	40.7%	9	33.3%	7	25.9%	27
5 (= highest)	17	48.6%	13	37.1%	5	14.3%	35
**Number of ED visits**	19	38.0%	15	30.0%	16	32.0%	50
0							
1	39	63.9%	14	23.0%	8	13.1%	61
2+	28	49.1%	14	24.6%	15	26.3%	57
**Number of primary care visits**	22	33.3%	23	34.8%	21	31.8%	66
1–10							
11–20	36	66.7%	10	18.5%	8	14.8%	54
21+	28	58.3%	10	20.8%	10	20.8%	48
**Number of inpatient visits**	56	77.8%	7	9.7%	9	12.5%	72
0							
1	22	39.3%	17	30.4%	17	30.4%	56
2+	8	20.0%	19	47.5%	13	32.5%	40
**Total**	86	51.2%	43	25.6%	39	23.2%	168

**Table 2:** Characteristics of patients with bipolar disorder identified in primary care (NYFHT) and hospital (NYGH) data, and those identified in both settings.

Patient characteristics	NYFHT		NYGH		NYGH and NYFHT		Total
	N	Percentage (%)	N	Percentage (%)	N	Percentage (%)	N
**Age range (years)**	94	72.3%	12	9.2%	24	18.5%	130
16–40							
41–60	94	81.0%	5	4.3%	17	14.7%	116
61+	97	78.2%	10	8.1%	17	13.7%	124
**Deceased**	281	78.3%	24	6.7%	54	15.0%	359
No							
Yes	4	36.4%	3	27.3%	4	36.4%	11
**Gender**	199	77.4%	20	7.8%	38	14.8%	257
F							
M	86	76.1%	7	6.2%	20	17.7%	113
**Number of co-morbidities**	149	74.9%	18	9.0%	32	16.1%	199
0–1							
2+	136	79.5%	9	5.3%	26	15.2%	171
**Income quintiles**	38	70.4%	7	13.0%	9	16.7%	54
Missing							
1 (= lowest)	41	75.9%	2	3.7%	11	20.4%	54
2	36	73.5%	4	8.2%	9	18.4%	49
3	40	76.9%	4	7.7%	8	15.4%	52
4	43	78.2%	5	9.1%	7	12.7%	55
5 (= highest)	87	82.1%	5	4.7%	14	13.2%	106
**Number of ED visits**	56	62.2%	15	16.7%	19	21.1%	90
0							
1	132	85.7%	5	3.2%	17	11.0%	154
2+	97	77.0%	7	5.6%	22	17.5%	126
**Number of primary care visits**	85	73.3%	12	10.3%	19	16.4%	116
1–10							
11–20	94	77.0%	8	6.6%	20	16.4%	122
21+	106	80.3%	7	5.3%	19	14.4%	132
**Number of inpatient visits**	176	90.7%	4	2.1%	14	7.2%	194
0							
1	80	66.7%	13	10.8%	27	22.5%	120
2+	29	51.8%	10	17.9%	17	30.4%	56
**Total**	285	77.0%	27	7.3%	58	15.7%	370

**Table 3:** Odds ratios for concordance among patients with schizophrenia and bipolar disorder. OR in bold are significant.

			Schizophrenia				Bipolar disorder			
Patient characteristics	Index Group	Reference Group	Odds ratio	95% confidence interval		P-value	Odds ratio	95% confidence interval		P-value
**Age range (years)**	**41–60**	**16–40**	**2.25**	**0.68**	**7.47**	**0.18**	**0.60**	**0.26**	**1.41**	**0.24**
	**61+**	**16–40**	**0.72**	**0.18**	**2.92**	**0.64**	**0.58**	**0.22**	**1.54**	**0.27**
**Gender**	**F**	**M**	**0.90**	**0.34**	**2.42**	**0.83**	**0.61**	**0.29**	**1.29**	**0.19**
**Income quintiles**	**2**	**1**	**0.83**	**0.17**	**4.03**	**0.81**	**1.24**	**0.42**	**3.64**	**0.70**
	**3**	**1**	**0.54**	**0.13**	**2.23**	**0.39**	**0.66**	**0.22**	**1.97**	**0.46**
	**4**	**1**	**0.80**	**0.22**	**2.92**	**0.74**	**0.76**	**0.25**	**2.31**	**0.62**
	**5**	**1**	**0.34**	**0.09**	**1.31**	**0.12**	**0.68**	**0.26**	**1.76**	**0.43**
**Number of co-morbidities**	**2+**	**0–1**	**0.64**	**0.20**	**2.06**	**0.45**	**1.40**	**0.62**	**3.16**	**0.42**
**Number of ED visits**	**1**	**0**	**0.35**	**0.08**	**1.51**	**0.16**	**1.19**	**0.46**	**3.03**	**0.72**
	**2+**	**0**	**0.89**	**0.26**	**3.03**	**0.85**	**1.51**	**0.63**	**3.66**	**0.35**
**Number of inpatient visits**	**1**	**0**	**3.04**	**0.78**	**11.78**	**0.11**	**3.99**	**1.64**	**9.68**	**0.002**
	**2+**	**0**	**2.42**	**0.64**	**9.20**	**0.19**	**8.38**	**3.16**	**22.22**	**< .0001**
**Number of primary care visits**	**11–20**	**1–10**	**0.20**	**0.06**	**0.68**	**0.01**	**1.39**	**0.59**	**3.25**	**0.45**
	**21+**	**1–10**	**0.32**	**0.10**	**1.09**	**0.07**	**0.81**	**0.33**	**1.97**	**0.64**

### Schizophrenia

We identified 168 patients with schizophrenia in the database (Table 1). The mean age was 50 years old. (SD 20.8; minimum 16, maximum 98) Of these, 86 were identified with this condition only in the NYFHT data (51.2%), and 43 only in NYGH data (25.6%). 39 patients (23.2%) had concordant diagnostic labels.

97 patients (57.7%) were women. 36.9% were age 40 or younger. 33.9% had two or more Emergency department visits and 23.1% had two or more hospital admissions during the three years of interest.

The odds ratios (ORs) of concordance were greater for patients with one inpatient admission (Table 3; OR 3.04, 95% CI 0.78 to 11.78 for one admission compared to none; P-value = 0.11). ORs of concordance were lower with higher income quintiles (OR among those in the highest income quintile vs. the lowest income quintile: 0.34; 95% CI 0.09–1.31, P-value = 0.12).

### Bipolar disorder

We identified 370 patients with bipolar disorder in the database (Table 3). The mean age was 51 years old. (SD 19.8; minimum 16, maximum 98) Of these, 285 were identified with this condition only in the NYFHT data (77.0%), and 27 only in NYGH data (7.3%). 58 (15.4%) of patients had concordant labels.

257 (69.5%) were women. 35.1% were age 40 or younger. 34.1% had two or more Emergency department visits and 15.1% had two or more hospital admissions during the three years of interest.

The ORs of concordance were greater for patients with one admission (Table 3; ORs 3.99, 95% CI 1.64 to 9.68 for one admission compared to none; P-value = 0.002) and two or more admissions (ORs 8.38; 95% CI 3.16–22.22 for those with 2+ inpatient visits vs. those with none; P-value<0.0001). There was no significant association between concordance and number of emergency room visits. ORs of concordance were slightly but not significantly lower with higher income quintiles, but this was not significant (OR among those in the highest income quintile vs. the lowest income quintile: 0.68; 95% CI 0.26–1.76; P-value = 0.43).

### Estimating the total population using capture-recapture modeling

**Fig 3:**
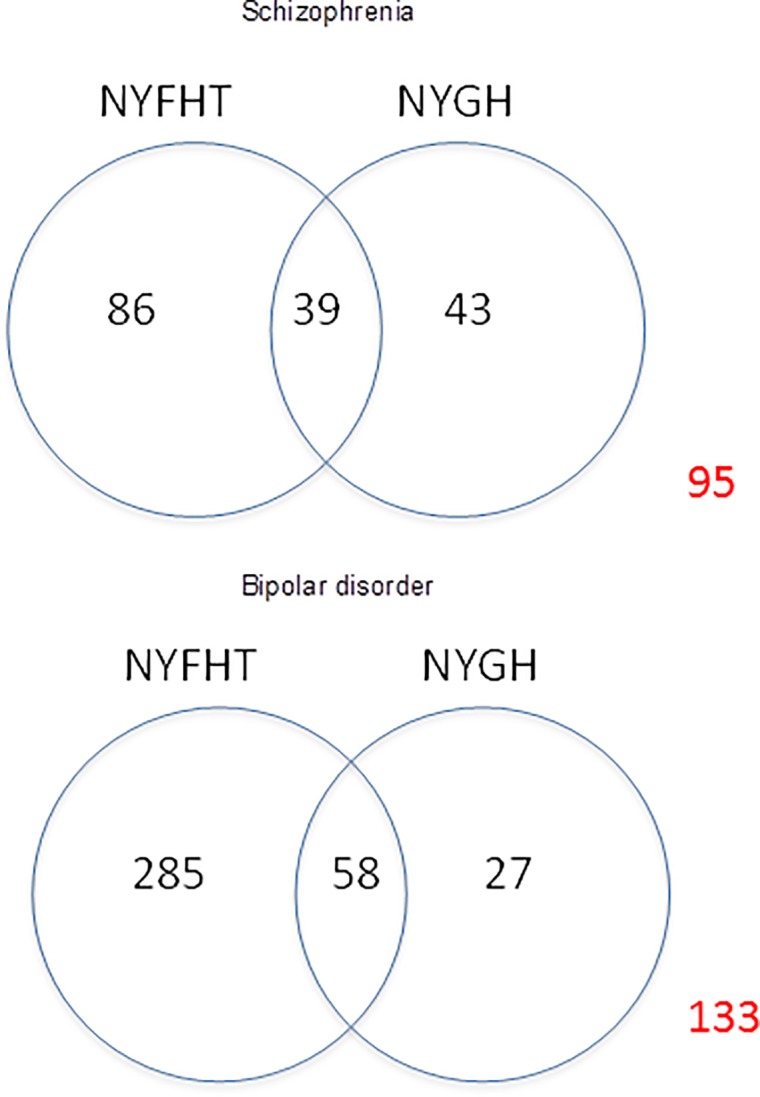
Estimate of total population of patients with schizophrenia and bipolar disorder in the Health Databank Collaborative database.

In order to estimate the total prevalence of these conditions between the two databases, we used capture-recapture modelling (Table 4). We estimated that 37.4% of the total patients with schizophrenia (95% CI 20.7–54.1) and 39.6% of total patients with bipolar disorder (95% CI 25.7–53.6) had missing diagnostic labels in NYGH and NYFHT while adjusting for patients’ age, sex, income quintiles and number of co-morbidities. Using these probabilities of missing labels, we estimated the presence of 268 schizophrenia patients (prevalence 259/100,000; 95% CI 212–366) and 613 bipolar patients (prevalence 592/100,000; 95% CI 498–798) in the Health Databank Collaborative database (Fig 3).

**Table 4:** Results of capture-recapture modelling to estimate population size of patients with schizophrenia and bipolar condition in NYGH and NYFHT.

Model	Proportion of Patients labeled in NYGH	Proportion of Patients labeled in NYFHT	Proportion of patients labeled in both	Proportion of Patients not labeled in either setting			Estimated Population size		
				Estimate	95% confidence interval		Estimate	95% confidence interval	
**Schizophrenia**									
**no covariate**	**0.256**	**0.512**	**0.232**	**0.361**	**0.254**	**0.467**	**263**	**225**	**315**
**age**	**0.253**	**0.512**	**0.234**	**0.357**	**0.249**	**0.464**	**261**	**224**	**314**
**age + sex**	**0.338**	**0.407**	**0.237**	**0.372**	**0.262**	**0.482**	**267**	**228**	**324**
**age + sex + income quintiles**	**0.300**	**0.408**	**0.251**	**0.337**	**0.212**	**0.461**	**253**	**213**	**312**
**age + sex + income quintiles + co-morbidities**	**0.310**	**0.419**	**0.227**	**0.374**	**0.207**	**0.541**	**268**	**212**	**366**
**Bipolar**									
**no covariate**	**0.073**	**0.770**	**0.157**	**0.264**	**0.174**	**0.354**	**503**	**448**	**572**
**age**	**0.073**	**0.772**	**0.155**	**0.267**	**0.177**	**0.358**	**505**	**449**	**577**
**age + sex**	**0.233**	**0.453**	**0.160**	**0.439**	**0.325**	**0.552**	**659**	**548**	**825**
**age + sex + income quintiles**	**0.223**	**0.451**	**0.166**	**0.420**	**0.295**	**0.546**	**638**	**525**	**815**
**age + sex + income quintiles + co-morbidities**	**0.200**	**0.451**	**0.168**	**0.396**	**0.257**	**0.536**	**613**	**498**	**798**

## Discussion

This study found substantial disagreement in diagnostic labeling of two SMIs–schizophrenia and bipolar disorder–between primary care and hospital records. Although there was higher agreement about diagnostic labeling among those with more frequent inpatient visits overall the study found overall poor concordance without any clear patterns to identify groups of patients that had good labeling agreement. Capture-recapture modeling suggested that between 35% to 37% of schizophrenic patients and 26% to 44% of bipolar patients may not have a label in either setting.

Previous work has identified low concordance for multiple conditions, including asthma and cardiovascular disease [27–28]. Our group has previously reported poor concordance (in the 20–30% range) for chronic obstructive pulmonary disease (COPD) and heart failure (HF) in the same database [20]. To our knowledge, there is no study exploring labeling concordance between primary care and hospital records for SMIs. This is surprising, given the essential role of primary care in preventing and managing physical illness among people with these conditions.

We were unable to conduct chart audits using established diagnostic criteria, such as OPCRIT for schizophrenia [29] in order to establish a ‘gold standard’ for determining diagnostic accuracy among study participants. It is likely there is some degree of over- and under- diagnosis in both settings, and that this may contribute to disagreement in diagnostic labeling.

The prevalence of schizophrenia in Canada is estimated to be between 0.2% - 2%. An estimate of 1% is commonly used [1]. The estimated prevalence in our study was 0.3% for schizophrenic patients and 0.6% for bipolar patients which is somewhat lower than this estimate This may be due to under diagnosis in the study population. In order for these diagnoses to be recorded, a clinician needs to document the diagnosis. Another possibility is that patients with serious mental illnesses in this population disproportionately access secondary care in specialized mental health settings, which are available in other institutions in the Greater Toronto Area, and access primary care through services that are geographically proximate to these mental health services. We are unable to test this hypothesis with our data.

A reason for the lack of concordance could be that these conditions were not identified in Emergency Department records; patients may have visited for minor conditions, and the SMI was never recorded by clinicians as part of the medical history in that setting. However, a substantial proportion of patients also had an inpatient admission in the three years of interest: 54.6% of patients with schizophrenia and 47.6% of patients with bipolar illness had been hospitalized at least once. Most patients admitted to hospital for mental health reasons are first seen in the emergency department. Patients may have been diagnosed with a substance use disorder in the ED and then subsequently identified as having SMI afterwards, which may not be reflected in these data. (The converse may also be true, where patients were diagnosed with SMI and subsequently identified as being under the influence of substances). Some patients were labeled with a SMI in the hospital system, were then seen by their family physician and were not labeled; this points to opportunities for cohort generation for quality improvement purposes in primary care in order for clinicians to fully document patients’ medical histories to ensure relevant conditions are identified and managed appropriately

We found that there were more patients with SMI labeled with schizophrenia or bipolar disorder only in primary care records. Many patients with these conditions are fairly stable, and may receive both physical and mental health care mostly through primary care providers. A study examining where people with mental illness access care in Alberta, Canada (another Canadian jurisdiction with provincially-funded, free at the point of care medicare) found that the majority of patients with mental illnesses are cared for primarily by primary care physicians [30]. In Ontario, there are incentives for physicians to identify patients with bipolar disorder and schizophrenia in the form of a lump sum yearly payment, for those who have provided care to 5 or more patients with these conditions. Enhancing these incentives may facilitate more complete documentation of these conditions in the primary care record.

### Limitations

There are several strengths of this study, such as its use of a novel linked primary-secondary care database, the exploration of health service access across the primary care-specialist care divide, and the extraction of fine granularity data from primary care EMRs. However, there are key limitations. As described above, we did not have access to the primary source of administrative data for inpatient mental health visits, the Ontario Mental Health Reporting System (OMHRS). Some patients may have been diagnosed with one of the conditions of interest during their inpatient hospitalization, after being seen in the emergency department, and this would not be reflected in available data. To mitigate this, we used sensitive definitions for these conditions. It is important to note that even from primary care data, the prevalence of these conditions was less than population estimates, suggesting veracity to our claim that there is a substantial proportion of patients for whom diagnostic labels are incorrect, even in primary care.

These data were obtained from a convenience sample of participating NYFHT physicians, rather than a representative sample. These practitioners differ from others by working in and around a major city, being younger and more interested in research than other Canadian family physicians [31]. This may affect the generalizability of these findings with respect to other family medicine settings such as rural and remote communities. The existence of a shared primary care-hospital research database at North York General Hospital is indicative of a close relationship between the hospital and local primary care providers; this relationship is not universal in the Canadian context. NYGH has a high degree of achievement in health information technology excellence, operating at Healthcare Information and Management Systems Society level 6. It won the Davies Enterprise award in 2016 for outstanding achievement of an organization which has utilized health information technology to substantially improve patient outcomes and value [32]. Whether this institutional emphasis on health information technology relates to improved documentation of diagnosis is unclear and it is not known the extent to which this affects generalizability.

Some discordant labeling may be due to improved diagnosis; for example, a patient may be initially diagnosed with ‘bipolar’ in the hospital record, but this is then updated to ‘major depressive disorder’ in the primary care record reflecting a more appropriate diagnostic label. Since data were only available from the emergency department and medical inpatient visits, it is possible that these conditions were simply not recorded during these visits. However, since these conditions are associated with such high morbidity and mortality, it is likely these conditions should have been documented in every assessment of their co-morbidities.

## Conclusions

We identified low concordance of diagnostic labeling for schizophrenia and bipolar disorder between primary care and hospital records. We also identified missing diagnostic labels across both settings. These are high-cost, high-risk conditions that are associated with frequent primary and secondary care visits, as well as frequent hospitalizations. It is essential to know in primary care if a patient has serious mental illness, because primary care is the key setting in which prevention takes place for conditions that are the largest contributors to mortality for these patients. Future research should identify ways to improve identification of these patients, and to improve diagnostic labeling.

## Supporting information

S1 Supporting Information Additional details regarding capture-recapture modelling. (PDF)
