# Supplementary material for: Agreement between primary care and hospital diagnosis of schizophrenia and bipolar disorder: A cross-sectional, observational study using record linkage
Source: PLoS One. 2019 Jan 7;14(1):e0210214. doi: 10.1371/journal.pone.0210214 (PMC6322753; doi:10.1371/journal.pone.0210214)
Supplement: S1 Supporting Information — (PDF) [file pone.0210214.s001.pdf]

Supporting Information S1: Additional details regarding capture-recapture modelling

The random-effect multinomial model was fitted to estimate:

(1) the probability of patients being looked after in the hospital (p_1); these were patients with a diagnostic label recorded in the hospital

(2) the probability of patients being looked after in primary care (p_2); these were patients with a diagnostic label recorded in primary care

(3) the probability of patients being looked after in both settings (p_3); these were patients with a diagnostic label recorded in both settings.

The model proposed by Tilling and Sterne (27) allowed us to estimate the probability that patients had a condition of interest, were seen in both settings and had their diagnostic label missed by both (p_o). This probability is based on the maximum likelihood estimator and can be explicitly estimated as:

$$p_o = \frac{1}{\left(1 + \frac{p_3}{p_1}\right) * \left(1 + \frac{p_3}{p_2}\right)}$$

In the absence of any covariate adjustment, the capture-recapture model uses two important assumptions: (1) the capture probabilities of the two sources (i.e. list of patients in each setting) are independent, (2) the probability of being captured from each source is assumed to be the same for every subject. However, these assumptions are often violated in many epidemiology studies, contributing towards under or over estimation of the population size. In a previous

study, we addressed this by stratifying the population estimate according to important patient characteristics: age range, gender, SES and co-morbidities.(15) In this study, we were unable to stratify the population estimate according to patient characteristics due to low sample sizes. Therefore, the accuracy of the population estimate may be impaired due to violation of these two assumptions.
